# Potentialities of a Membrane Reactor with Laccase Grafted Membranes for the Enzymatic Degradation of Phenolic Compounds in Water

**DOI:** 10.3390/membranes4040678

**Published:** 2014-10-06

**Authors:** Vorleak Chea, Delphine Paolucci-Jeanjean, José Sanchez, Marie-Pierre Belleville

**Affiliations:** Institut Européen des Membranes (IEM), ENSCM, UM2, CNRS, Université de Montpellier 2, CC 047, Place Eugène Bataillon 34095, France; E-Mails: vorleak.chea@gmail.com (V.C.); delphine.paolucci@iemm.univ-montp2.fr (D.P.-J.); jose.sanchez@iemm.univ-montp2.fr (J.S.)

**Keywords:** enzymatic membrane reactor, laccase, phenolic compounds, wastewater treatment

## Abstract

This paper describes the degradation of phenolic compounds by laccases from *Trametes versicolor* in an enzymatic membrane reactor (EMR). The enzymatic membranes were prepared by grafting laccase on a gelatine layer previously deposited onto α-alumina tubular membranes. The 2,6-dimethoxyphenol (DMP) was selected  from among the three different phenolic compounds tested (guaiacol, 4-chlorophenol and DMP) to study the performance of the EMR in dead end configuration. At the lowest feed substrate concentration tested (100 mg·L^−1^), consumption increased with flux (up to 7.9 × 10^3^ mg·h^−1^·m^−2^ at 128 L·h^−1^·m^−2^), whereas at the highest substrate concentration (500 mg·L^−1^), it was shown that the reaction was limited by the oxygen content.

## 1. Introduction

Due to the high toxicity of phenolic compounds for living organisms, their removal from industrial or domestic wastewaters is a current and very important issue. Among the different available technologies for wastewater treatment, the enzymatic conversion of phenols into quinones using laccases EC 1.10.3.2 (benzenediol:oxygen oxidoreductases) can be revealed as an attractive alternative [1,2,3,4,5]. These enzymes are multi-copper proteins, which are capable of oxidizing a variety of phenolic and non-phenolic compounds requiring only the presence of oxygen [6]. Laccases catalyze one-electron oxidations by transferring electrons from four substrate molecules to one molecule of molecular oxygen, which is reduced to water [7]. Reaction products–phenoxy radicals–undergo further radical reactions generating various oligomers and polymers, which can be easily eliminated from effluents by sedimentation or filtration.

The coupling of membrane technology and enzymatic reactions in an enzymatic membrane reactor (EMR) results on a process intensification allowing the filtration of charged solutions like wastewaters, simultaneously, with an enzymatic conversion. Two types of EMRs are described in the literature: enzymes may be either circulated freely on the retentate side or immobilized on the membrane surface or inside its porous structure [8]. In the first configuration, the reaction takes place in solution and the membrane allows recycling the biocatalyst, whereas, in the second configuration, the reaction takes place during the transfer through the membrane, allowing high conversion rates because the contact between the substrate and the biocatalyst is enhanced. Both EMR types have been already used for the removal of phenolic compounds from wastewaters [9,10,11,12,13,14,15].

In this work, we chose to study the second type of EMR with immobilized laccase because it has been reported that the immobilized form of this enzyme is much more stable than the free form [16,17,18]. Concerning the fouling phenomena, we took in consideration the assumption that oxidation products (polymers) will be formed after the transmembrane transfer. Active membranes were elaborated by covalent immobilization of laccase from *Trametes versicolor* onto α-alumina membranes with 0.2 µm, 0.8 µm, and 1.4 µm of mean pore diameter. The choice of an inorganic porous support allows a complete active layer removal thanks to a classical cleaning procedure when the biocatalyst becomes inactive, permitting to use again the support for fresh enzymes grafting [19,20]. The main objective of this work is to study and to describe the potentiality of this active grafted membrane towards phenolic compounds degradation.

The efficiency of this enzymatic membrane towards three different model phenolic compounds (4-chlorophenol, guaiacol and 2,6-dimethoxyphenol (DMP)) was evaluated. These compounds were chosen since they may be present in some wastewaters. Guaiacol and DMP are representatives of lignin and humic acid degradation products [21] and chlorophenol is widely used as a fungicide or bactericide; it is also a breakdown product of a wide variety of pesticides.

The enzymatic reactions were first carried out in an EMR in a batch configuration (*i*.*e*., tangential filtration mode with both retentate and permeate recycling). In a second step, DMP degradations were carried out using a continuous dead-end filtration configuration and the study was particularly focused on the effects of substrate feed flux and substrate concentration on the reaction conversion.

## 2. Experimental Section 

### 2.1. Enzyme and Chemicals

A commercial laccase powder from *Trametes versicolor* (53739-1G-F; Sigma-Aldrich, St Quentin Fallavier, France) was used as bio-catalyst; it was stored at −20 °C until required for used. Substrate solutions were prepared from: 2,2-azino-bis(3-ethylbenzothiazoline-6-sulfonic acid) (ABTS) (11557; Fluka, St Quentin Fallavier, France), 4-chlorophenol (185787-100G; Sigma-Aldrich), guaiacol (50880-100G; Fluka) and DMP (38772-25G; Fluka) in 50 mM citrate-phosphate buffer 0.1 mM of CuSO_4_, pH 4. The gelatine (24,360,290; Prolabo, Labover, Montpellier, France), glutaraldehyde (G6257; Sigma-Aldrich) and enzyme solutions were prepared in 50 mM phosphate buffer at pH 7 and 20 °C.

### 2.2. Preparation of the Active Membranes

The active membranes were prepared according to a three-step procedure described in a previous paper [14]. Firstly, ceramic supports (α-alumina tubular membranes from Pall-Exekia, France-length: 15 cm, external diameter: 1 cm, internal diameter: 0.7 cm, effective area: 28.6 10^−4^ m^2^ and mean pore size: 0.2 μm, 0.8 μm or 1.4 μm) were coated with a gelatine layer by filtration of a 10 g·L^−1^ aqueous gelatine solution (transmembrane pressure ΔP = 200 kPa and tangential velocity u = 2 m·s^−1^ at 20 °C) for 30 min. The thin polymer layer obtained was activated by filling the membranes with a glutaraldehyde solution (4% (w/v) for 1 h at room temperature. Finally, a laccase solution (10 g·L^−1^ of commercial powder in 50 mM phosphate buffer, pH 7) was allowed to react with the free aldhehyde groups of the glutaraldehyde for 2 h at room temperature. After each step, the excess solution was removed by washing the membranes four times with the same phosphate buffer. The enzymatic membranes were then dried in a desiccator under P_2_O_5_ and stored in a desiccator under silica gel until required for use. For blank experiments, non-active membranes were prepared using a similar procedure, but the enzyme solution was replaced by a buffer solution without enzyme.

After use, the ceramic supports were soaked in a sodium hypochlorite solution (5% (v/v)) prepared with warm water (40 °C) for 15 min. The membranes were then rinsed and washed, following a standard regeneration sequence that involves successive basic and acidic washings, as recommended by the supplier. The ceramic supports were not re-used until the initial water flux was recovered.

### 2.3. Enzymatic Membrane Reactor (EMR)

**Figure 1:**
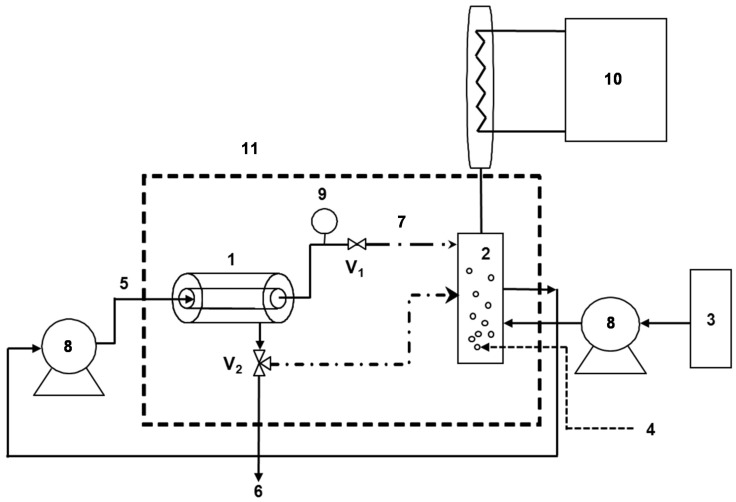
Pilot unit. **1**: membrane module; **2**: feed tank; **3**: fresh substrate feed; **4**: air bubbling feed; **5**: substrate feed; **6**: permeate exit; **7**: retentate; **8**: pump; **9**: pressure transducer; **10**: cooling system; **11**: temperature controlled chamber; **V_1_** and **V_2_**: valves.

The pilot and the EMR used for phenols degradation are presented in Figure 1. The reactor can be used in two distinct configurations: dead-end and tangential, by respective closing or opening of valve V_1_. A controlled temperature chamber was used to maintain the EMR at 40 °C. In dead-end configuration, the substrate concentration of the feed solution remains constant throughout the experiment, whereas, in batch tangential configuration (*i*.*e*., both retentate and permeate are recycled in the feed tank), the system works in non-steady state conditions while substrate concentration decreases continuously with time. If required, permeate can be recycled into the feed tank through valve V_2_. In the continuous experiments carried out over a long period (in dead-end configuration mode without recycling of the permeate stream), fresh substrate was continuously added to the feed tank. Continuous flow of air (5 L·h^−1^) was bubbled through the feed solution maintaining the reaction medium under oxygen saturation as verified by an oxygen sensor (inoLab^®^ Oxi 730, supplier SODIPRO, Echirolles, France) and the contents were mixed perfectly using a magnetic stirrer. A cooling system comprising a condenser programmed at 5 °C was placed over the air exit to avoid water evaporation and phenolic compound loss.

### 2.4. Phenolic Compounds Degradation in the EMR

Before use, each enzymatic membrane was placed in the EMR and hydrated with 50 mM citrate-phosphate buffer containing 0.1 mM of CuSO_4_ at pH 4. The effect of the type of substrate was studied in batch configuration with the EMR being operated in tangential filtration mode with a transmembrane pressure of 200 kPa and a tangential velocity of 0.16 m·s^−1^. The reactor was filled with a solution of one of the three phenolic compounds prepared at 100 mg·L^−1^ in the same buffer described above. Both the retentate and permeate were recycled into the feed tank. The remaining experiments were carried out exclusively with DMP solutions in continuous dead-end filtration mode (valve V_1_ closed and permeate being continuously removed). Different concentrations of DMP (from 100 to 500 mg·L^−1^ prepared in the same citrate-phosphate buffer pH 4) were tested. Feed flow (F) varied from 0.03 to 0.36 L·h^−1^, corresponding to 11 to 128 L·h^−1^·m^−2^. These values were chosen based on the mean pore diameters of the membranes (0.2 μm, 0.8 μm and 1.4 μm). Depending on the feed flow the transmembrane pressure values varied between 0 kPa and 400 kPa. For each experiment, both permeate and feed samples were withdrawn regularly for HPLC analysis. All of the experiments were carried out at 40 °C.

To take into account possible non-enzymatic degradation, blank experiments were also carried out with the membranes being prepared without enzyme for both filtration modes and all tested substrates.

Substrate conversion for tangential filtration mode was calculated using Equations (1) and substrate conversion and consumption for continuous dead-end filtration mode were calculated using Equations (2) and (3):


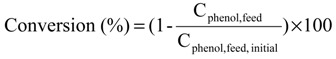
(1)


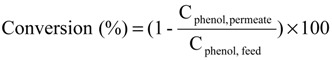
(2)


Consumption rate (mg.h^−1^) = (C_phenol,feed_ – C_phenol,permeate_) × F (3)

where F is the feed flow (L·h^−1^); and C_phenol,feed,initial_, C_phenol,feed_ and C_phenol,permeate_ are the substrate concentrations (mg·L^−1^) in feed at time “0” and at time “t” and permeate at time “t”, respectively.

Each run was carried out with a new enzymatic membrane and the results shown below are the mean values of the various experiments.

### 2.5. Analysis

Laccase activity was determined from the change in optical density (at 420 nm, ε_420_ = 36,000 M^−1^·cm^−1^) of 50 mM citrate-phosphate buffer, pH 4 containing ABTS (1 mM) as substrate [22]. ABTS was chosen since this substrate is more sensitive than phenol based substrate [23]. One unit of laccase activity was defined as the amount of enzyme required to oxidize 1 µmol of ABTS per minute.

The immobilization yield was estimated by comparing the activity of the laccase solution before and after the immobilization step, taking into account the activity of washings, as well as that of the permeate recovered at the early stages of the conditioning step (see Section 2.4). The concentration in phenolic compounds of the withdrawn samples were analyzed by HPLC (Waters e2695 Separations Module, Waters^®^, France) using a Symmetry^®^ C_18_ column. Samples of 20 μL were injected and eluted at 1 mL·min^−1^ (mobile phase: 50 mM citrate-phosphate buffer, pH 4/acetonitrile (50/50) in the case of 4-chlorophenol and guaiacol or 50 mM citrate-phosphate buffer, pH 4/acetonitrile (80/20) for 2,6-dimetnoxyphenol) and 30 °C. Elution profiles were monitored at 296 nm (Detector UV/VIS (PDA 2996), Waters^®^, St Quentin en Yvelines, France).

## 3. Results and Discussion

### 3.1. Degradation of Phenolic Compounds in the EMR

**Figure 2:**
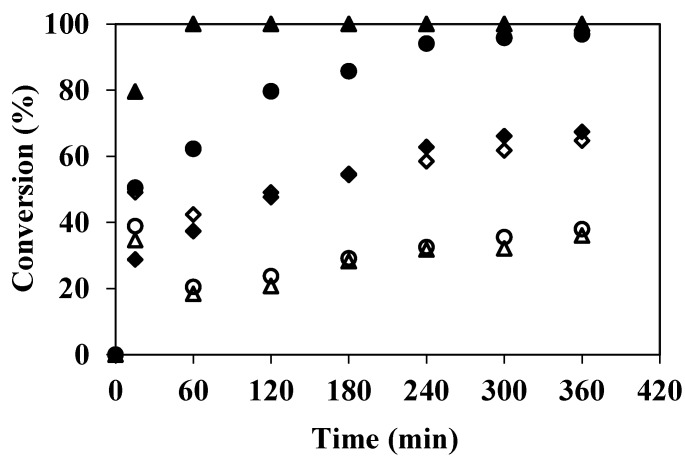
Conversion of 4-chlorophenol (◊,♦), guaiacol (○,●), 2,6-dimetoxyphenol (∆,▲) degradation in EMR working in batch mode (without enzyme-open symbols-and with enzyme-closed symbols-, 100 mg·L^−1^ of phenolic substrate in 50 mM citrate-phosphate buffer pH 4 with 0.1 mM of CuSO_4_, ΔP = 200 kPa, tangential velocity = 0.16 m·s^−1^, T = 40 °C, membrane pore diameter 0.2 μm). The error of measurements is ≤10%.

The catalytic efficiency of an enzymatic process depends to a very large extent on the choice of the reactor, the reaction media, the operating conditions and of course, the reactivity of the enzymes towards the substrates. In the case of laccases, the degree of conversion of phenolic compounds depends on the chemical structure of the compounds. Although, laccases from *Trametes versicolor* are able to degrade a wide range of simple and complex phenolic compounds [4,24,25], it is important to characterize the EMR under good measurable operating conditions by selecting a substrate with quantifiable reactivity to laccase action. As explained above, these preliminary experiments were carried out in batch tangential configuration (in tangential filtration mode with recycling both the retentate and permeate into the feed tank). The pH and temperature were fixed to 4 and 40 °C, respectively, these values correspond to the optimal activity towards ABTS. Figure 2 shows the results evolution of the conversion *versus* time obtained for the three phenolic compounds studied (4-chlorophenol, guaiacol and DMP) during batch experiments carried out with or without enzymes. It is important to notice that the average immobilization yield of laccase obtained for the membranes used in this work was equal to 8.5% ± 1.5%. This yield corresponds to an amount of laccase immobilized on membranes equal to 7.0 × 10^4^ ± 5.0 × 10^3^ U·m^−2^. This immobilization yield could not be defined in terms of immobilized proteins since the possible release of gelatin during the grafting procedure could lead to an overestimation of the protein concentration resulting on miss estimation of the total laccase grafted.

Figure 2 shows that, after 6 h, the conversion observed in blank runs (membranes non-grafted with laccase) was 65% for 4-chlorophenol, but it was only 38% and 36% for guaiacol and DMP, respectively. A spontaneous partial depletion of phenolic compounds from aqueous solutions was already observed by Ko and Chen [14], Ba *et al*. [26], Arsuaga *et al*. [27], Williams *et al*. [28], and Bódalo *et al*. [29]. According to these authors, spontaneous depletion might due to one more phenomena related to substrate molecular weight, substrate adsorption, filtration operating conditions, or auto-oxidation. In particular, enzymatic depletion can take place simultaneously with auto oxidation, but generally it is impossible to distinguish the depletion level caused by each one of these reactions. In our case, as both permeate and retentate were recycled in the feed tank, intermediate products could react with the substrate. On the contrary, when enzymatic membranes were used, conversion was complete (100%) for guaiacol and DMP, whereas no change in conversion was observed for 4-chlorophenol. According to this result, we cannot assert that the laccase is not active towards 4-chlorophenol, but only that the EMR does not enhance the depletion level observed by auto-degradation. Furthermore, it can be noticed that DMP was completely depleted after less than 1 h of reaction, whereas conversion of guaiacol reached 100% only after 4 h under the temperature (40 °C) and pH (4) tested. Nevertheless, it must be taken into account that in batch configuration, permeate and retentate are recycled continuously and, therefore, conversion results from cumulative depletion of the concentration. The results shown in Figure 2 are in good agreement with the conversions reported in some previous works. Indeed, chloro substituents have been reported to be much less reactive to laccases than methoxy substituents [30,31]. Among the monochlorophenols, the 4-chlorophenol is known to be recalcitrant to some laccases [32]. Nevertheless, these results are not exactly in good agreement with the work of Lante *et al*. [11] who used laccase immobilized onto a spiral-wound asymmetric polyethersulphone and reported that even if the DMP was the most reactive phenol, the 4-chlorophenol was more reactive than guaiacol. Ko and Chen [14] have reported that the oxidation of methoxy- and hydroxy-substituted phenols by laccase was enhanced by the formation of rather stable free radicals. Additional electron-donating methoxy groups in DMP provide more free-radicals containing resonance forms for reaction intermediates. Chivukula *et al*. have also published that the number of methoxy substituent was very important, lacasse was much more reactive towards the 2,6-dimethoxy-subsituted compounds than to 2-methoxy-substituted compounds [31].

As it has been described above, the study of the effect of process parameters on the degradation rate was carried out exclusively with DMP in continuous dead-end filtration mode. The values of pH and temperature were the same as previously and corresponded also to the optimal for DMP degradation with the laccase from *Trametes versicolor*. To take into account the auto-oxidation degree, some experiments were carried out with non-active membranes. In such configuration the DMP auto-degradation was not observed as it was noticed in batch configuration. The feed concentration remained practically constant and equal to the initial concentration throughout the experiment. Hence, it confirms that the non-enzymatic oxidation observed during experiments in batch configuration could be the result of operating conditions (*i*.*e*., substrate agitation, shear stress due to tangential velocity, recycling of the oxidation products, *etc*.). Indeed, DMP consumption was always calculated by comparing DMP concentrations in feed and permeate samples withdrawn at the same time.

### 3.2. Feed Flux Effect on DMP Degradation

**Figure 3:**
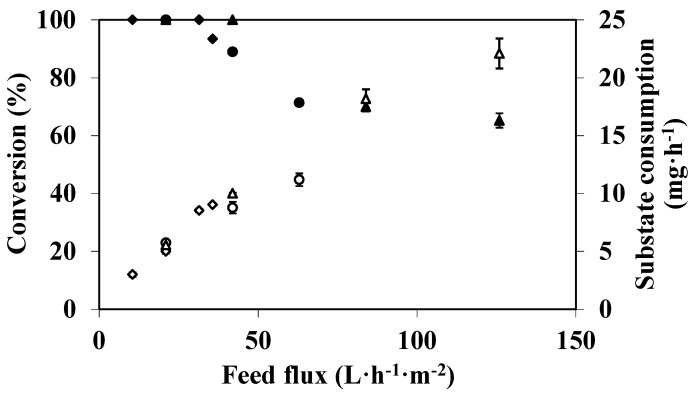
Feed flux effect on DMP conversion (closed symbols) and substrate consumption (open symbols) in an EMR used in a continuous dead-end filtration configuration (100 mg·L^−1^ DMP in 50 mM citrate-phosphate buffer pH 4 with 0.1 mM CuSO_4_, T = 40 °C, membrane pore diameter: 0.2 µm ((◊, ♦), 0.8 µm (○, ●), and 1.4 µm (∆, ▲).

When an EMR is used in dead-end filtration mode, the feed or substrate flux and permeate flux are identical. Thus, increasing this parameter results in a decrease in residence time, which may affect reactor efficiency and particularly the conversion. In order to study the effect of feed flux on DMP degradation, experiments were carried out with different feed fluxes and enzymatic membranes prepared with different mean pore diameters of the raw ceramic support (0.2, 0.8, and 1.4 μm). All the experiments were carried out for 90 min. Substrate consumption and conversion *versus* substrate flux results are shown in Figure 3.

When feed flux is lower than 32 L·h^−1^·m^−2^, substrate consumption is the same for all membranes for a given feed flux, and does not depend on the mean pore size of the membrane support. It was not possible to draw conclusions above 42 L·h^−1^·m^−2^ as the experiments could not be carried out with the 0.2 µm and 0.8 µm porous supports because transmembrane pressure increases continuously and reached values higher than 400 kPa. Figure 3 also shows that any additional supply of substrate resulting from an increase in feed flux produces consumption enhancement. The substrate consumption increases from 2.7 mg·h^−1^ to 22.2 mg·h^−1^ with an increase in feed flux from 11 L·h^−1^·m^−2^ to 128 L·h^−1^·m^−2^. This result is consistent with those reported by Lante *et al*. [12] and Erhan *et al*. [13], and confirms the assumption that the reaction is substrate-limited under the studied conditions. At the highest feed flux (128 L·h^−1^·m^−2^), 7.9 × 10^3^ mg·h^−1^·m^−2^ DMP were depleted from a feed solution containing 100 mg·L^−1^ of DMP. It should be noted that this DMP consumption rate does not seem to be the optimum since, according to the figure trend, higher values of DMP consumption can be expected at higher feed fluxes. Although it is not always easy or reasonable to compare the performances of various EMRs, enzymes and/or substrates can be different, it should be noted that the value reached is similar to/or higher than the consumption reported in the literature for the degradation of other phenolic compounds in EMRs. Erhan *et al*. [13] reported for a feed of 9 × 10^−4^ mL·cm^−2^·s^−1^ (*i*.*e*., 32.4 L·h^−1^·m^−2^) of 467 mg·L^−1^ of a catechol solution a degradation rate of 0.153 µg.s^−1^·cm^−2^ (*i*.*e*., 5.5 × 10^3^ mg·h^−1^·m^−2^. From the values reported by Lante *et al*. [11], it is possible to estimate the DMP degradation capacity of their EMR at 60 mg·h^−1^·m^−2^. Despite of a relatively high membrane surface area (0.14 m^2^), their DMP removal efficiency was only equal to 69% when the reactor was fed at 2 mL.min^−1^ (*i*.*e*., 0.86 L·h^−1^·m^−2^) with 100 mg·L^−1^ of substrate solution.

Finally conversion was affected when feed flux was increased but it can be maintained at 100% as long as the feed flux is kept below 32 L·h^−1^·m^−2^. An increase in feed flux leads to rapid conversion decrease because we decrease simultaneously the contact time, for example, for fluxes of 64 L·h^−1^·m^−2^ and 128 L·h^−1^·m^−2^, conversion reaches only 75% and 65%, respectively. Consequently, if total phenolic compound degradation is required, the feed flux should not exceed 32 L·h^−1^·m^−2^. Increasing of feed flux reducing the conversion was already observed by Lante *et al*. [11] and Lloret *et al*. [33] when they studied, respectively, the degradation of phenolic compounds by laccase immobilized onto a spiral-wound asymmetric polyethersulphone membrane and by laccase immobilized in a microreactor.

Alternatively, it is also possible to maintain a high consumption rate with a good conversion by using a multi-step process involving EMRs with different active surfaces, placed in series, for example, a first series of EMRs can be fed at high feed flux, which ensures high phenolic compound consumption, while a second series is fed with the permeate of the first EMRs at a lower feed flux, allowing a complete degradation of the phenolic compounds.

### 3.3. Effect of Substrate Concentration on DMP Degradation

**Figure 4:**
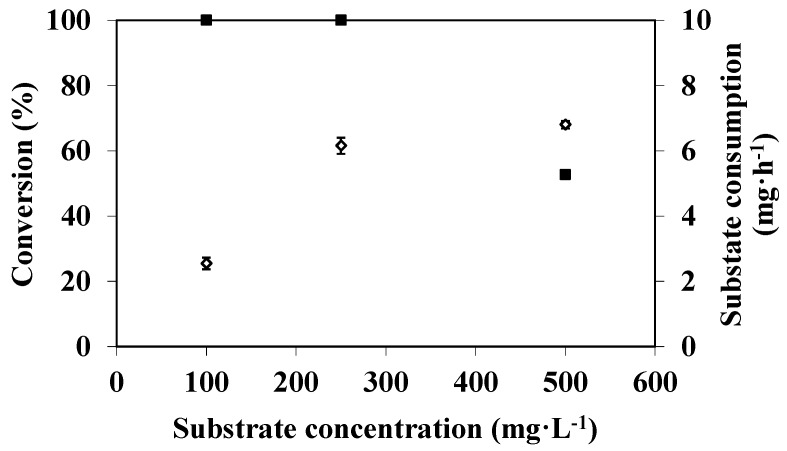
Substrate concentration effect on DMP degradation in an EMR used in a continuous dead-end filtration configuration (100, 250, and 500 mg·L^−1^ DMP in 50 mM citrate-phosphate buffer pH 4 with 0.1 mM CuSO_4_, feed flux 11 L·h^−1^·m^−2^, T = 40 °C, membrane pore diameter: 0.2 μm) (substrate consumption (◊); conversion (■).

According to the above results, the efficiency of the EMR was limited by a lack of substrate when it was fed with a 100 mg·L^−1^ of DMP solution, irrespective of the feed flux used. In order to overcome this limitation, experiments were carried out with an enzymatic membrane prepared with a 0.2 µm porous support and with substrate solutions prepared at different concentrations (100, 250, and 500 mg·L^−1^). The feed flux was kept constant at 11 L·h^−1^·m^−2^ throughout a 90 min operating time. The conversion and the substrate consumption values *versus* substrate concentration are shown in Figure 4.

Firstly it can be seen that conversion remains constant and equal to 100% as long as the substrate concentration is less than or equal to 250 mg·L^−1^ and then it drops to 46% when substrate concentration increases up to 500 mg·L^−1^. In the latter case, a large amount of substrate was transferred across the membrane before being transformed. Nevertheless, substrate consumption is enhanced from 2.4 mg·h^−1^ to 6.2 mg·h^−1^ when the concentration is increased from 100 to 250 mg·L^−1^; it then tends to level off, increasing very slightly to 6.9 mg·h^−1^ when the substrate concentration reaches 500 mg·L^−1^. Katuri *et al*. [34] also showed that the phenolic compounds were completely degraded by laccase until a concentration of 20 mg·L^−1^ whereas at higher concentrations the conversion decreased.

**Figure 5:**
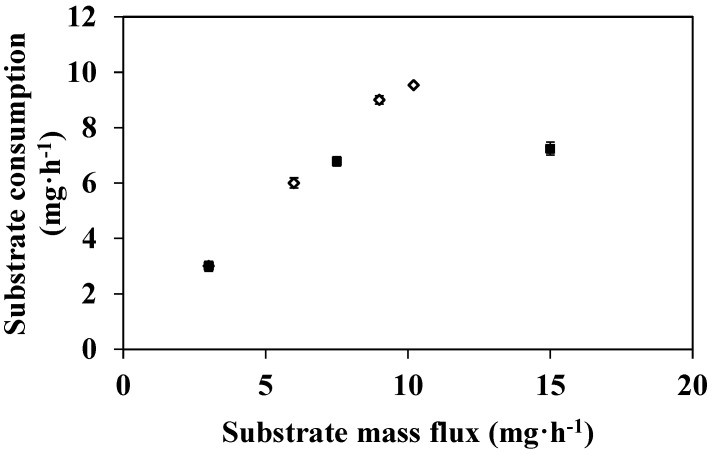
Substrate mass flux effect on DMP degradation in an EMR used in a continuous dead-end filtration configuration (DMP in 50 mM citrate-phosphate buffer pH 4 with 0.1 mM CuSO_4_, T = 40 °C, membrane pore diameter: 0.2 μm) (reactor fed with 100 mg·L^−1^ DMP at flow rate increased (◊); reactor fed at flow rate constant 11 L·h^−1^·m^−2^ with 100, 250 and 500 mg·L^−1^ DMP (■)).

If we compare these last results with those obtained at different feed fluxes but with a constant substrate concentration (*i*.*e*., 100 mg·L^−1^) (see Figure 5). We can notice that increasing the mass flux of the substrate by either increasing feed flux or substrate concentration results on different substrate consumption. In the first case, consumption increases continuously and the maximum consumption rate observed is equal to 9.5 mg·h^−1^ for a substrate mass flux of 10.5 mg·h^−1^ (*i*.*e*., when the reactor was fed at 36.5 L·h^−1^·m^−2^ with a 100 mg·L^−1^ of DMP solution). In the second case, the consumption levels off and the maximum consumption rate is only 6.9 mg.h^−1^ for a substrate mass flux of 15.7 mg·h^−1^ (*i*.*e*., when the reactor was fed at 11 L·h^−1^·m^−2^ with a 500 mg·L^−1^ of DMP solution). In this last case, the real substrate concentration near the catalytic site would be certainly important, whereas it would tend to zero in the first set of experiments. Indeed according to Figure 3, the conversion rates obtained with 0.2 µm membrane are always equal or close to 100%, whereas this parameter decreases until 50% when the reactor was fed at low feed flow with high substrate concentration. In the first case, the reaction is, thus, limited by a lack of DMP whereas, in the second case, when DMP concentration is higher than 250 mg·L^−1^ (*i*.*e*., 1.6 × 10^−3^ M) and then the reaction may be limited either by the substrate inhibition or by a lack of O_2_.

It is well known that high substrate concentrations which normally increase the velocity of enzyme reactions can cause inhibition in about 20% of all known enzymes. However, to our knowledge this phenomenon has not been reported for the couple laccase-DMP. Nevertheless, laccases activity has been reported to be strongly dependent on oxygen concentration. According to Riva [35], laccase theoretically catalyze the conversion of four molecules of phenolic compounds, together with the reduction of one molecule of oxygen to two molecules of water. However, Kurniawati and Nicell [36] reported that the molar ratio between phenol transformed and oxygen consumed in the catalytic reaction is not constant and varies from 1 to 4 when the phenol concentration increases in the reaction mixture. At high DMP concentration (*i*.*e*., 1.6 × 10^−3^ M or 246 mg·L^−1^), if the stoichiometric ratio is assumed to be equal to 4, the O_2_ concentration should be at least 4 × 10^−4^ M. However, even if the air flow was kept constant for all our experiments (*i*.*e*., 5 L·h^−1^), which allowed the reaction mixture to remain statured as verified by controls, at 40 °C the oxygen concentration was 6.4 mg·L^−1^ or 2 × 10^−4^ M of O_2_. This value is much lower than required, thus, the reaction was probably limited by the lack of O_2_.

In view to confirm this hypothesis, we decided to increase the O_2_ concentration of the feed solution. For this purpose, the total pressure in the feed tank was increased from 100 to 250 kPa while the temperature was kept constant and equal to 20 °C. In such conditions the O_2_ concentration measured was then increased from 8.5 mg·L^−1^ to 21 mg·L^−1^. The degradation rates observed were equal to 7.3 ± 0.7 mg·h^−1^ and 14.5 ± 2.3 mg·h^−1^ for experiments carried out at 100 and 250 kPa, respectively, all the other operating conditions being kept similar. These results confirm that an increase in oxygen content leads to an increase of reaction rate.

## 4. Conclusions 

Enzymatic membranes were successfully prepared by covalent bonding of *Trametes versicolor* laccases onto an α-alumina tubular porous support previously coated with a protein. Among the three phenolic compounds tested, 2,6-dimethoxyphenol (DMP) resulted to be the most reactive substrate for the laccase grafted membranes tested in an enzymatic membrane reactor (EMR) operating in tangential filtration configuration. For EMR operating in dead-end filtration configuration, we found that DMP depletion increased continuously with the feed flux. A substrate depletion of 7.9 × 10^3^ mg·h^−1^·m^−2^ were observed when the reactor was fed continuously with a 100 mg·L^−1^ of DMP solution at 128 L·h^−1^·m^−2^. These first results present attractive perspectives for the treatment of industrial phenolic wastewaters. However, investigations are required in order to improve the process.

At high DMP concentrations (over 250 mg·L^−1^), the degradation seems to be limited by the lack of oxygen. Furthermore, if substrate flow-rate is too high, substrate conversion will not be complete and some phenolic compound can remain in the permeate stream. However, in such cases, a bundle of membranes with an in series/parallel configuration with aeration steps can be designed in order to reach a complete conversion of the effluent while obtaining a filtrated water depleted from the phenolic compounds. The degradation of phenolic compounds in wastewaters needs the determination of substrates-enzyme affinity and the process has to be optimized for each type of substrate. Nevertheless, the direct application of this process to domestic wastewaters should not be considered because the organic matrix of such effluents can contain different types of organic molecules able to react with laccases. This process seems to be more indicated for the treatment of charged phenolic industrial wastewaters.

A work at very low concentrations (less than 1 ppm) and with real wastewaters is under way in order to examine the feasibility of this enzymatic technology under real environmental conditions.
